# Effects of cover cropping on orchard soil microbiomes: Mechanisms and perspectives

**DOI:** 10.3934/microbiol.2026010

**Published:** 2026-04-30

**Authors:** Guan Guan, Pingzhi He, Yu Miao, Gaofeng Zhou, Guidong Liu

**Affiliations:** National Navel Orange Engineering Research Center, Gannan Normal University, Ganzhou 341000, China

**Keywords:** Cover cropping, soil microbiome, rhizosphere, carbon-nitrogen-phosphorus cycling, soil health

## Abstract

Orchards have long faced severe soil erosion, acidification of red soils, low nutrient-use efficiency, and frequent soil-borne diseases. Conventional clean tillage combined with intensive chemical inputs often fails to simultaneously improve fruit yield and quality while safeguarding orchard ecological security. Cover cropping (i.e., managed groundcover vegetation) introduces persistent surface plant cover and introduces continuous inputs of root exudates, litter, and residues, while simultaneously modifying soil moisture, temperature, aggregation, porosity, and nutrient availability. Consequently, it reorganizes the soil microbiome from the rhizosphere scale to community-network scales and drives key ecological processes such as carbon sequestration, nitrogen and phosphorus turnover, and disease suppression. Mechanistically, cover crops (i) enhance the supply of labile carbon through root exudation and residue return, stimulating microbial assimilation and enzyme-mediated decomposition and promoting SOC stabilization via microbial necromass formation-mineral association/aggregate protection; and (ii) optimize microbial habitats by improving aggregate architecture, pore structure, and water-holding capacity, and by regulating pH and nutrient availability, thereby increasing the abundance and functional potential of key guilds (e.g., diazotrophs, nitrifiers/denitrifiers, and microorganisms involved in organic-P mineralization) and their functional gene repertoires. In addition, cover cropping may strengthen system stability and suppressiveness through multi-trophic interactions and reconstruction of the soil micro-food web. However, under drought conditions or during the juvenile-tree stage, trade-offs can emerge due to context-dependent tree–groundcover competition for water and nutrients. Future progress requires long-term field experiments integrating multi-omics, isotope tracing, and process-based flux measurements to establish causal evidence chains and scenario-specific models linking management–microbial mechanisms–ecosystem services. Developing operational microbiome-based indicators will provide a scientific basis for groundcover species selection, cover pattern optimization, and fertilizer reduction with improved efficiency, as well as disease mitigation and fruit-quality enhancement.

## 1. Introduction

Orchards are important perennial production systems widely distributed across diverse agroecological regions worldwide. In many regions, including Mediterranean olive-growing areas and the Gannan navel orange production region in southern China, orchards are often established on sloping land or other environmentally sensitive sites [1]–[3]. These systems contribute substantially to regional ecology and socioeconomic development, but they also face persistent soil constraints, including erosion, compaction, nutrient depletion, and biodiversity loss, especially under long-term intensive floor management [4]–[6]. Although clean tillage can suppress weeds in the short term, prolonged clean tillage often reduces soil fertility and biological diversity and may ultimately impair fruit yield and quality [7]. Against this background, cover cropping has emerged as a sustainable orchard floor management strategy. By introducing specific cover species or conserving beneficial natural groundcovers, it helps build a more balanced tree–groundcover system and creates opportunities to improve soil quality and ecosystem functioning [8]. Depending on implementation, orchard groundcover management can be classified into sown and natural cover. Common sown covers include legumes such as *Trifolium pratense* and grasses such as *Lolium perenne* L., which differ in nitrogen fixation, residue quality, and decomposition dynamics, thereby exerting distinct effects on soil nutrient supply, soil structure, and microbial activity [9],[10]. Through root traits, litter and residue inputs, and root-exudate chemistry, cover crops regulate soil carbon and nitrogen accumulation, aggregate formation, and other key soil processes, thus providing an important basis for microbiome-mediated changes in orchard ecosystems.

The soil microbiome is widely regarded as the core engine of soil ecosystems, and its functional stability underpins soil fertility, ecosystem service delivery, and terrestrial material balance. Soil microorganisms contribute to aggregate formation, water retention, organic matter transformation, and N–P–K cycling [11]. Some actinomycetes and fungi form hyphal networks that physically entangle soil particles and secrete polysaccharide glues that bind microaggregates into stable macroaggregates [12], thereby increasing aggregate stability and enhancing soil water retention. Through diverse metabolic pathways, microorganisms transform organic carbon into more stable compounds (e.g., lipids, polysaccharides, and recalcitrant organic matter). They also secrete extracellular enzymes (e.g., cellulases and ligninases) to decompose plant and animal residues and convert rhizodeposits into inorganic carbon, low-molecular-weight organic acids, and mineral salts [13]. Moreover, the soil–plant–atmosphere nitrogen cycle is primarily driven by microbial functional guilds: diazotrophs convert atmospheric N₂ into bioavailable nitrogen, whereas nitrification and denitrification regulate rhizosphere inorganic-N transformations and N losses as N₂/N₂O, jointly determining plant N supply and environmental impacts [14]. Likewise, phosphate-solubilizing microorganisms mobilize sparingly soluble P by secreting organic acids, protons, and phosphatases, increasing available P; and potassium-solubilizing microorganisms enhance K release and availability, improving rhizosphere N-P-K accessibility and nutrient supply [15],[16]. In hilly and mountainous orchards, management practices such as clean tillage, cover cropping, and mulching reshape soil microbial community structure and diversity by altering organic matter inputs and nutrient/hydrothermal microenvironments, and these shifts can be linked to changes in soil properties and fruit quality [17].

**Figure 1.**
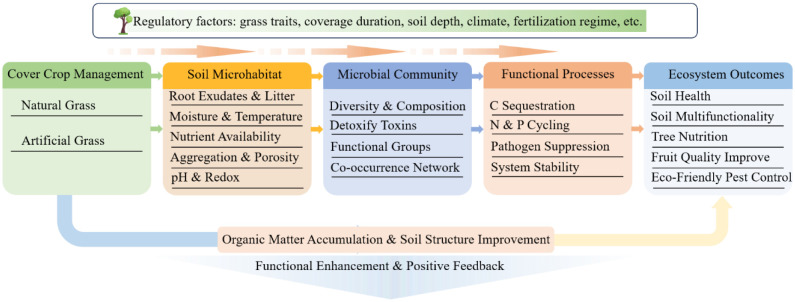
Conceptual framework linking cover-crop management to soil microenvironment, microbial community, functional processes, and orchard ecosystem services. Note: Cover-crop management (e.g., cover type, species composition, spatial arrangement, mowing/residue return, and management intensity) modifies the soil microenvironment (C inputs, moisture-temperature regime, nutrient availability, aggregation/porosity, and pH/redox), which in turn reshapes microbial community attributes (diversity, composition, functional guilds, keystone taxa, and co-occurrence network properties). These microbial shifts regulate key functional processes, including carbon sequestration, nitrogen and phosphorus cycling, pathogen suppression, and system stability/resilience, ultimately contributing to enhanced ecosystem services such as soil health and multifunctionality, tree nutrition and stress resilience, improved yield/fruit quality, and eco-friendly pest and disease control. Regulating factors (plant traits, cover duration, soil depth, climate, and fertilization regime) modulate the strength and direction of these pathways. Dashed arrows indicate positive feedback mediated by organic matter accumulation and soil structure improvement.

Recent studies on orchard cover cropping have gradually moved beyond simple descriptions of microbial diversity and community composition toward a more process-oriented understanding of how cover crops regulate microbiome assembly and ecosystem functioning. In addition to reporting shifts in dominant taxa and network structure, increasing attention has been paid to rhizosphere filtering by root-derived compounds, microbial carbon-processing pathways, necromass-mediated carbon stabilization, functional guild differentiation, and multi-trophic interactions within the soil food web [17]–[19]. At the methodological level, advances in metagenomics, metatranscriptomics, rhizosphere metabolomics, stable-isotope tracing, and process-based flux measurements are beginning to provide stronger evidence for linking cover-crop management with microbial functions and soil ecosystem services. Understanding microbiome responses and mechanisms under cover cropping is therefore crucial for optimizing orchard management and enhancing ecosystem services. To provide an overview of the conceptual logic discussed in this review, the main pathways linking cover-crop management, soil microenvironment, microbial community shifts, and orchard ecosystem services are summarized in Figure 1.

This review first summarizes how cover cropping reshapes orchard soil microbial community structure and diversity. Second, we review microbiome-mediated effects on carbon turnover and stabilization, nutrient cycling, disease suppression, and soil multifunctionality. Third, we discuss core interactions within the plant–soil–microbiome continuum, including root exudate filtering, habitat modification, and multi-trophic interactions. Finally, we outline current limitations and future research priorities for establishing causal links between cover-crop management, microbiome assembly, and ecosystem services.

## 2. Effects of cover cropping on soil microbial community structure and diversity in orchards

Cover cropping persistently increases belowground carbon inputs (root exudates, litter, and residues) while concurrently modifying soil physicochemical conditions on slopes (organic matter accumulation, aggregate structure and aeration, soil moisture dynamics, and nutrient availability). These combined effects reshape microhabitats and biotic interactions in both the rhizosphere and bulk soil. As a result, the composition and diversity of bacterial and fungal communities in orchard soils undergo systemic shifts, with turnover in dominant taxa and clearer functional differentiation. Meanwhile, structural properties of microbial co-occurrence networks—such as connectivity, modularity, and robustness—may also change with management, thereby influencing the stability of soil ecological processes and the resistance of orchard systems to disturbance.

### 2.1. Bacterial community responses

The impacts of cover cropping on soil bacterial communities in orchards are mainly reflected in increased diversity and pronounced structural differentiation, and these effects are jointly driven by cover species traits, soil depth, and management duration.

Castellano-Hinojosa et al. reported that, compared with the grower-standard row-middle management, cover crops significantly increased soil microbial alpha diversity and reshaped bacterial community structure in commercial citrus orchards in Florida, USA [20], suggesting that cover cropping helps form more diverse and complex bacterial communities. In a three-year field experiment in an apple orchard in Tai'an, Shandong (a hilly region), inter-row cover crops (*Trifolium pratense*, *Bouteloua gracilis*, *Eragrostis trichodes*, *Eragrostis curvula*, and *Elymus canadensis*) generally increased bacterial richness and α-diversity, with dominant phyla mainly comprising Proteobacteria and Acidobacteria; importantly, effects differed markedly among cover species [21]. Such species effects are typically linked to plant inputs and rhizosphere processes: legumes (e.g., clovers and alfalfa) can enhance soil N supply via symbiotic N fixation and alter bacterial community structure through combined exudate and litter inputs, whereas grasses (e.g., ryegrass) often supply more cellulose-rich substrates and modify pore structure/microhabitats, thereby enriching cellulose-degrading taxa (e.g., *Bacillus*) [22],[23]. In addition, root exudates contain diverse organic acids and amino acids that serve as carbon sources and signaling molecules, recruiting and stimulating the proliferation of specific rhizosphere bacteria (e.g., *Sphingomonas*), which can further amplify cover species–driven differentiation of community composition and functions [24].

Vertically, cover-cropping effects on bacterial communities exhibit a clear topsoil effect. Because carbon inputs from exudates and litter are concentrated in the surface layer, changes in community composition and diversity are typically most pronounced in the 0–20 cm layer. As depth increases to 20–40 cm or even 40–60 cm, differences often diminish, consistent with the general pattern of decreasing bacterial richness/diversity with depth [17],[25]. Across 0–60 cm profiles, bacterial abundance is often higher under cover cropping than under clean tillage, but significant differences are usually confined to the 0–20 cm (or extended to 0–40 cm) layer, whereas deeper layers respond weakly [22],[26]. This pattern reflects the fact that most roots and rhizosphere processes of both trees and groundcovers are concentrated in upper soil layers; as depth increases, reduced substrate inputs and lower baseline microbial abundance/activity make community responses slower and less detectable [27],[28].

Long-term cover cropping (≥3 years) can shift bacterial communities from dominance by r-strategists toward K-strategists, which is often interpreted as a signal of increasing ecosystem maturity. R-strategist bacteria (e.g., *Bacteroidota*) reproduce rapidly and efficiently exploit pulsed resources and thus tend to dominate frequently disturbed, pulse-fed soils under clean tillage. In contrast, K-strategists (e.g., *Acidobacteriota*) are better adapted to stable resource supply and lower stress. Zhang et al. showed that alfalfa cover in a sandy loam apple orchard in Shanxi, China, provided sustained carbon inputs (via litter decomposition and root exudation) and improved soil physicochemical properties (e.g., lower bulk density and higher organic matter) [10], creating favorable conditions for K-strategists and enhancing microbiome stability and resistance to disturbance.

### 2.2. Fungal community dynamics

Compared with bacteria, fungal responses to cover cropping are more guild-specific and temporally dynamic, and the core effects are typically dual: enrichment of beneficial fungi and suppression of pathogens.

Relative to clean tillage, cover cropping often increases fungal biomass (e.g., fungal PLFA) and promotes fungal α-diversity, largely because continuous inputs from exudates, fine-root turnover, and litter residues provide stable carbon substrates, while improved aeration and organic matter in topsoil enhance microhabitat conditions [10],[29]. Effects differ among cover types. In a multi-year study in a semi-arid vineyard in the United States, Rocha et al. found that interrow cover crops increased microbial network complexity and fungal-to-bacterial ratios, indicating strengthened belowground microbial interactions and greater system connectedness under cover cropping [30]. Another study in a Shanxi sandy loam apple orchard reported that alfalfa cover increased microbial diversity and network complexity, with legumes showing particularly strong effects on building tighter and more modular networks [10]. In a hillside vineyard in central Italy, Silvestroni et al. reported that legume-enriched cover crops (*Trifolium alexandrinum*) improved grape yield and fruit quality attributes, suggesting that legume-based cover management can enhance production performance in perennial orchard systems [31]. Besides, in karst mango orchards, legume covers (e.g., *Desmodium intortum*, *Chamaecrista rotundifolia*, and *Vicia villosa* Roth) increased bacterial, fungal, and arbuscular mycorrhizal fungal (AMF) biomass, enriched *phoD*-associated microorganisms, and strengthened bacterial–fungal co-occurrence, highlighting strong reshaping of interaction patterns by legumes [32]. By contrast, grasses often produce more cellulose- and lignin-rich residues, which favor saprotrophic fungi capable of decomposing complex substrates, thereby shifting fungal community composition in ways linked to litter quality and input rates [33]. Overall, differences in exudate chemistry and residue quality (e.g., N-rich legume substrates vs. fiber-rich grass substrates) regulate fungal niche partitioning and drive differential abundances of AMF and saprotrophic guilds, influencing carbon cycling and organic matter transformation in orchard soils [34].

Cover cropping can substantially alter the functional structure of orchard fungal communities. This reassembly is reflected not only in increased network connectivity and complexity but also in a redistribution between pathogenic and beneficial guilds. By increasing organic matter and sustaining carbon inputs (litter return and rhizodeposition), cover crops improve soil microenvironments that favor saprotrophic or symbiotic fungi and tend to suppress some potential pathogens (e.g., *Fusarium*) [29],[35]. Root exudates contain chemical signals and nutrients that selectively attract or inhibit taxa, driving a functional shift from pathogen-dominated assemblages toward saprotrophic/symbiotic-dominated ones, thereby enhancing plant health and soil suppressiveness [34],[36].

In orchard ecosystems, cover-cropping effects on fungi often show a *short-term suppression–long-term promotion* pattern. During the initial 1–2 years, competition between groundcovers and tree roots for water and nutrients can transiently reduce microbial activity or constrain changes in fungal biomass [37],[38]. With extended management (often >3 years), cumulative inputs of root residues, litter, and exudates provide a more stable resource base and habitat, promoting SOC and total N accumulation and improving aggregate structure and water retention [33],[39]. Empirical evidence reports that alfalfa cover can increase SOC in topsoil by ~25% or more [10].

### 2.3. Microbial network complexity and stability

Beyond altering community composition and diversity, cover cropping can rewire microbial interactions (mutualism, competition, antagonism). In a vineyard study on Hanford-type soils in the United States, Rocha et al. reported that bacterial–fungal molecular ecological networks became more complex under cover crops (e.g., *Phacelia* spp. and rye), with more bacterial–fungal links; bacterial–bacterial increases were more competitive, whereas fungal–fungal increases were more cooperative, and bacteria–fungi synergistic relationships also increased [30]. In general, higher network complexity and robustness often co-occur with increased soil organic matter, microbial biomass, and N fertility indicators, implying more active microbially mediated decomposition and nutrient transformation that favor nutrient accumulation [40],[41]. High-throughput sequencing and co-occurrence network analyses further show that, relative to clean tillage (CT), cover cropping can expand network size and topological complexity. For example, in orchard systems, legume cover (alfalfa) increased node numbers by ~40% and edge numbers by ~55% compared with CT, while enhancing modularity, connectivity, and evenness of degree distribution, indicating a more stable and robust network with tighter potential interactions [10]. Such complex and robust networks may reflect stronger metabolic complementarity and collaboration across taxa and may improve community resistance and recovery after disturbances such as drought. Drought-focused studies suggest that cover crop–induced root channels and related mechanisms can shift rhizosphere bacteria toward more stress-tolerant strategies and antioxidant/energy-saving metabolic pathways, helping maintain functions under water stress and accelerating recovery [42],[43].

The increase in network complexity is largely attributed to the greater diversity of carbon inputs induced by cover cropping. By introducing living groundcovers, orchards receive higher organic C inputs and develop more stratified canopy-understory structures [38]. Different cover species provide distinct carbon substrates via species-specific exudates and litter composition [44]. Legumes often release amino acids and carboxylic acids (e.g., proline, glutamate, citrate, and malate) and some secondary metabolites, which can support diazotrophs and promote mutualisms with rhizobia and AMF, strengthening C-N coupling [27],[45],[46]. Grasses more commonly release soluble sugars and produce residues rich in structural carbon (cellulose/hemicellulose; cellulose may account for ~35%–50% of plant dry mass), favoring saprotrophic fungi and cellulose-degrading bacteria [45],[47]. As cover-crop configurations shift from monocultures to mixtures (e.g., multi-species sowings), networks often transition from simple and loose to complex and stable (more nodes/edges, higher connectivity and robustness), which may increase soil resistance and resilience to external disturbances [29],[48],[49].

**Table 1.** Structural responses of soil microbiomes to orchard cover cropping: bacteria, fungi, and co-occurrence networks.

Cover-cropping context	Bacterial community response	Fungal community response	Co-occurrence network response	Main drivers (soil/environment)	Ref.
Cover crop vs. clean tillage	•Bacterial richness and α-diversity↑vs. clean tillage •Community composition shifts	•↑fungal biomass/α-diversity •Higher saprotroph/symbiont representation, lower potential pathogens	•Network complexity and robustness ↑ •More positive associations	•Increased C inputs •Improved aggregation and moisture buffering •Nutrient availability	[10],[20],[29],[31],[33]
Legume cover	•Recruitment of N-associated taxa; α-diversity↑ •Readily available substrates	•Potential symbionts↑and beneficial guilds •Pathogen suppression signals may strengthen	•May enhance positive associations •Depend on the fertilization background and moisture	•Biological N and labile C inputs •pH buffering and changes in available P	[26],[32],[46]
Grass cover	•Richness/α-diversity ↑ •Long-term tends to favor K-selected community features	•Often↑fungal diversity with more residue inputs •More saprotroph-related activity	•Network complexity associated↑with higher C-input and aggregation	•Litter and fibrous root inputs; SOC accumulation; aggregate stability; moisture regulation	[20],[21],[22],[44]
Mixture	•↑bacterial diversity and functional redundancy •Compositional divergence across depths	•Redistribute fungal guilds; potential↑beneficial fungi •Outcomes may vary with mixture composition and climate	•↑positive associations and network complexity with higher plant diversity/mixtures	•Resource heterogeneity and spatial niche partitioning •Stable microhabitats via improved soil structure	[23],[48],[49]
Topsoil vs. subsoil (depth effect)	•Topsoil effect: stronger diversity shifts in 0–20 cm •Subsoil responses slower	•Fungal effects often concentrated near residue/exudate inputs	•Network complexity typically declines with depth	•Declining labile C, oxygen, and root influence with depth •Changes in pH, moisture, and nutrient stratification	[10],[25],[26],[28]

## 3. Microbial functional regulation and ecological effects under cover cropping

### 3.1. Microbially mediated carbon turnover and pathways of SOC stabilization

Soil organic carbon (SOC) retention involves key steps, including enzyme-mediated depolymerization, microbial assimilation and transformation, and stabilization/preservation. Soil microorganisms and their extracellular enzymes are central biological drivers of organic matter turnover and SOC formation/stabilization [50]–[52]. Microbes can assimilate plant-derived carbon and transform it into microbial products and residues (e.g., cell-wall components, lipids, and polysaccharides) that more readily enter stable carbon pools. Microbially derived organic matter, especially microbial necromass, has been recognized as a major contributor to SOC and may account for more than half of SOC in many ecosystems [53],[54]. Notably, the stock of necromass carbon can be tens of times larger than living microbial biomass carbon (on the order of ~40-fold), underscoring its importance for SOC accumulation and persistence [51]. Functional differences between bacteria and fungi are also important: bacteria often preferentially exploit soluble, easily decomposable substrates, whereas fungi are more effective in degrading complex structural compounds, such as lignin and cellulose, and play key roles in aggregate formation and stabilization [55]–[57]. Fungal hyphae and metabolites promote particle enmeshment and aggregation, enhancing physical protection of SOC within aggregates and increasing SOC stabilization potential [56],[58]. From a management perspective, cover crops and residue/organic amendments typically increase microbial diversity and reshape community structure. Substrate chemistry (e.g., soluble C fraction and lignocellulose content) influences microbial assembly and carbon fate. An increasing body of evidence indicates that SOC increases are closely linked to necromass accumulation; in cover-crop systems, SOC gains are often realized through necromass—particularly fungal necromass—entering mineral-associated organic matter pools [59],[60].

Cover crops influence carbon turnover and stabilization mainly by altering both the quantity and quality of carbon entering soil. Labile compounds released through root exudation can rapidly stimulate microbial growth and enzyme production, thereby accelerating the early depolymerization and assimilation of plant-derived carbon [61]. At the same time, repeated inputs of fine-root residues and aboveground biomass provide structurally complex substrates that favor fungal decomposition and promote the conversion of plant carbon into microbial necromass. This distinction is important because the fate of carbon under cover cropping is not simply determined by greater carbon input but by whether that carbon is preferentially mineralized, incorporated into microbial biomass, or retained as necromass, subsequently protected within aggregates or mineral-associated organic matter [62]. Recent work showed that cover-crop root exudates can redirect soil microbiome functional trajectories [24], while orchard studies further demonstrated that legume cover crops enhanced SOC partly through increased microbial necromass accumulation, especially fungal necromass in mineral-associated fractions [63].

Extracellular enzymes catalyze SOM mineralization, and their sources and activities depend on microbial abundance and composition, while also being regulated by substrate supply and soil conditions [64]. Structural carbon in exogenous organic inputs (e.g., straw and green-manure residues) is dominated by cellulose and hemicellulose, so carbon turnover largely relies on hydrolase systems involved in cellulose/hemicellulose degradation [65]. Key hydrolases commonly used to represent C acquisition include β-glucosidase, cellobiohydrolase/cellobiosidase, and β-xylosidase [66]. Cellulose is typically cleaved by endoglucanases into oligosaccharides; cellobiohydrolases then release cellobiose from chain ends, and β-glucosidase hydrolyzes cellobiose to glucose. This cooperative endo–exo/chain-end release–terminal hydrolysis mechanism is a classic model of cellulase systems [67]. β-Xylosidase participates in the terminal hydrolysis of xylan in hemicellulose and, together with endoxylanases, releases monosaccharides such as xylose, facilitating hemicellulose decomposition and utilization [65],[68]. Accordingly, increased exogenous carbon inputs often stimulate microbial biomass and enzyme activities, accelerating decomposition and organic-matter turnover. Tracking enzyme dynamics, therefore, provides mechanistic insights into SOC turnover and stabilization after organic inputs. Numerous studies show that straw return, incorporation of green manures, and organic fertilization can improve microbial habitats by increasing labile organic C, supplementing N and other nutrients, and improving structure, thereby altering communities and increasing the abundance of functional microbes and multiple extracellular enzyme activities [66],[69],[70]. Importantly, extracellular enzymes are not always free in soil; some enzymes are adsorbed onto clays, Fe/Al oxides, or humic substances, forming mineral-bound enzymes that affect enzyme longevity, apparent activity, and substrate accessibility [71],[72]. Studies also indicate that increases in cellulase activity often track changes in dissolved organic carbon release and decomposition, supporting cellulases as indicators of the intensity of cellulose degradation and carbon turnover rates [73],[74]. Saccà et al. further reported a positive relationship between β-glucosidase (or its encoding genes) and SOC content, supporting its use as a sensitive indicator for SOC accumulation/stabilization-related processes [75].

### 3.2. Microbial regulation of nitrogen and phosphorus cycling: key guilds and functional genes

By increasing rhizosphere carbon inputs and improving microenvironments, cover cropping often reshapes the composition and functional potential of nitrogen-cycling microorganisms in orchard soils. Compared with clean tillage, cover crops can increase the abundance of ammonia-oxidizing archaea/bacteria and their key functional genes (e.g., *amoA*), and alter denitrifier communities (e.g., taxa carrying *nirK* and *nosZ*), thereby influencing nitrification–denitrification coupling and nitrogen-loss risks, including N₂O emissions. Zhang et al. showed that in kiwifruit orchards, cover cropping with white clover significantly increased the abundances of AOA-*amoA* and AOB-*amoA* [76]. In Florida citrus orchards, Castellano et al. found that legume–nonlegume mixtures increased gene abundances related to N fixation and nitrification, increased *nosZ*-type denitrifiers, and reduced N₂O emissions [77]. Recent metagenomic analyses in peach orchards with inter-row cover (white clover and perennial ryegrass) also showed that cover cropping enhanced nitrification-related genes (e.g., *amoA* and *hao*) and the functional potential of multiple N transformation pathways [78]. In parallel, legumes can contribute additional nitrogen inputs through symbiotic N fixation, increasing soil-available nitrogen pools [79].

In phosphorus cycling, cover cropping can stimulate P-solubilization and organic-P mineralization through increased rhizosphere carbon inputs and residue return, and can enhance phosphatase systems (e.g., alkaline/acid phosphatases encoded by *phoD*/*phoX*/*phoA*) that hydrolyze organic P and improve P bioavailability [80],[81]. In studies conducted in lemon orchards in South Africa and yellow-soil citrus orchards in Sichuan, China, it was found that, compared with herbicide-based weed control, white-clover cover significantly increased soil N and P and phosphatase activity and enriched functional genes related to oxidative phosphorylation, purine/pyrimidine metabolism, and transport processes, suggesting enhanced microbial capacity for nutrient acquisition and metabolism [82],[83]. In citrus orchards, *phoD*-harboring functional guilds and their network complexity are closely associated with phosphatase activity and available P, supporting the pathway *microbially mediated organic-P mineralization → increased nutrient availability* as a key mechanism by which cover crops improve P cycling [84]. In tree-based systems, more broadly, green manures/cover crops can enhance P-cycling enzyme activities and microbial functions, thereby increasing labile P pools and promoting tree P uptake [85].

Cover cropping can also increase nutrient turnover efficiency by enhancing enzyme activities related to nutrient cycling. Many studies report increased activities of urease, sucrase, and β-glucosidase under cover crops [19],[22]. Such enzyme increases indicate heightened microbial metabolic activity and accelerated conversion of nutrients from organic to inorganic forms, thereby improving plant-available nutrient supply.

### 3.3. Microbiome-driven disease suppression, soil multifunctionality, and fruit-quality benefits

From a biocontrol perspective, cover cropping often increases soil organic matter and microbial diversity and strengthens the connectivity and stability of microbial networks, enhancing natural soil suppressiveness and reducing the risk of soil-borne diseases. Grassi et al. reported that, in a Mediterranean olive orchard, cover crop management increased bacterial diversity and, over time, promoted greater arbuscular mycorrhizal fungal richness and more positive connections within AMF networks, suggesting enhanced belowground network connectivity under cover cropping [86]. Similar observations have been reported in hilly apple orchards in China, where cover crops increased microbial diversity/abundance and enzyme activities and shifted community composition toward profiles more favorable for nutrient cycling and system health [21]. Broadly, cover crop–driven microbiome restructuring can enrich antagonistic or plant growth–promoting taxa (e.g., *Bacillus* and *Trichoderma*) and their functional complementarity, while reducing the relative abundance of potential pathogens (e.g., *Fusarium*), providing a stronger biological barrier and resilience for tree rhizospheres [35],[87],[88].

In terms of soil health, cover cropping can enhance soil multifunctionality by reshaping microbial communities and networks and by improving the system's buffering capacity against disturbances. Changes in microbial diversity and composition can influence nutrient retention, organic matter transformation/stabilization, and biodegradation of pollutants [89]. In a four-year groundcover experiment in Chinese hickory (*Carya cathayensis*) plantations, Wu et al. reported that intercropping rape (*Brassica rapa* L.), perennial ryegrass (*Lolium perenne* L.), and Chinese milk vetch (*Astragalus sinicus* L.) significantly increased microbial biomass C and improved carbon-substrate utilization activity (AWCD) and metabolic diversity (Shannon and evenness) measured by Biolog EcoPlate [90]. Long-term cover-crop management has also been shown to increase soil multifunctionality and shift microbial networks toward more complex and stable structures [48].

**Figure 2.**
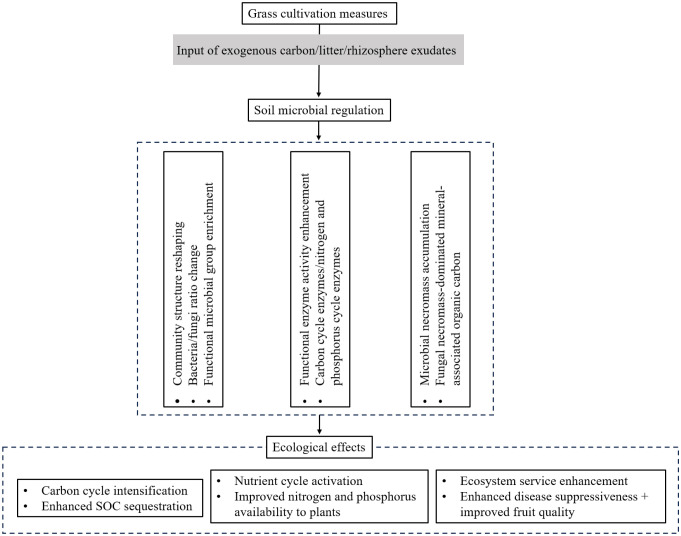
Framework of soil microbial regulation and ecological effects driven by grass cultivation. Note: This figure illustrates the chain relationship: grass cultivation measures regulate soil microorganisms from three dimensions (community structure reshaping, functional enzyme activity enhancement, microbial necromass accumulation) via inputting exogenous carbon, litter, and rhizosphere exudates, which further drive three categories of ecological effects: carbon cycle intensification (enhanced SOC sequestration), nutrient cycle activation (improved nitrogen and phosphorus availability to plants), and ecosystem service enhancement (enhanced disease suppressiveness and improved fruit quality).

Cover cropping can additionally influence tree nutrient acquisition, stress resistance, and fruit-quality formation indirectly via changes in rhizosphere carbon supply, soil microenvironments, and microbiome functions. In a hillside vineyard in central Italy, Silvestroni et al. reported that legume-enriched cover crops, including an annual *Trifolium alexandrinum* treatment and a perennial legume–grass mixture, improved grape yield and fruit quality attributes, suggesting that legume-based groundcover management can positively affect production performance in perennial orchard systems [31]. In a vineyard study in Portugal, Teixeira et al. found that different native-plant cover crop mixtures, particularly treatments featuring grasses and legumes, differentially shaped soil and berry microbiota, indicating that cover-crop identity can influence fruit-associated microbial assembly and thus potentially contribute to quality-related responses through a microbiome-mediated pathway [91]. Mechanistically, many beneficial rhizosphere microorganisms can facilitate nutrient acquisition (N fixation, P/K solubilization), secrete phytohormones, and induce systemic resistance, jointly regulating plant growth and quality-related metabolism [92],[93]. The major pathways by which cover cropping regulates soil microbial functions and associated ecological effects are summarized in Figure 2.

## 4. Core interactions within the plant–soil–microbiome continuum

### 4.1. Tree–groundcover interactions and root exudate–driven microbiome assembly

One central pathway by which cover cropping reshapes orchard soil microbiomes is through changes in the quantity, composition, and persistence of root-derived carbon inputs. Both fruit trees and groundcovers continuously release exudates into the rhizosphere, thereby modifying local carbon supply, pH, and redox conditions and creating chemically distinct microsites for microbial recruitment and activity. Root exudates include not only sugars, amino acids, and organic acids that serve as readily available substrates but also a wide range of specialized metabolites, such as phenolic acids, flavonoids, and terpenoids, which function as biochemical signals mediating chemotaxis, colonization, quorum-sensing interference, and antagonistic interactions [36],[94],[95]. Therefore, the influence of cover cropping on the microbiome is not merely a consequence of greater carbon input but also of altered chemical selectivity in the rhizosphere. Recent studies suggest that this chemically mediated selectivity can drive functional differentiation of orchard microbiomes under different cover-crop systems. Long-term bristlegrass cover, for example, significantly increased SOC, SON, and multiple carbon-cycling enzyme activities in orchard rhizosphere soils, while also shifting microbial community structure and diversity, indicating that sustained rhizosphere carbon inputs can expand microbial niche space and stimulate functional guilds involved in carbon and nutrient turnover [96]. Importantly, different cover crops may recruit microorganisms through distinct exudate signatures. In legumes, flavonoids and isoflavonoids released by species such as white clover act as key rhizobial signaling molecules that induce *nod* gene expression and promote symbiotic associations, thereby favoring nitrogen-related microbial functions [97]. In grasses, specialized metabolites such as benzoxazinoids can impose stronger biochemical filtering on rhizosphere bacteria by favoring tolerant or metabolically adapted taxa and are increasingly recognized as important determinants of microbiome structure and disease-suppressive potential [98],[99]. More broadly, plant-driven rhizosphere filtering is now understood to be a major mechanism underlying the assembly of disease-suppressive microbiomes, as exudate chemistry can selectively enrich beneficial taxa while disfavoring potential pathogens [100]. Taken together, these findings indicate that cover-crop effects on orchard microbiomes should be interpreted not simply as changes in diversity or composition but as a process of exudate-driven ecological selection that links cover-crop identity with microbiome assembly, functional differentiation, and plant health outcomes.

At the spatial scale, niche separation between tree and groundcover roots in depth and horizontal position generates heterogeneity in carbon inputs (exudates and fine-root turnover) and microenvironmental conditions across soil layers and root domains, driving vertical and horizontal differentiation of microbial communities [101]. Studies conducted in corn and soybean fields in Iowa, USA, showed that microbial richness and diversity generally decrease with depth, and that topsoil is more sensitive to vegetation inputs and management [102]. Column experiments in wheat further suggest that root architecture can shift the depth distribution of microbial hotspots and influence microbial biomass and extracellular enzyme activities across depths [103]. In orchard systems, a Belgian apple orchard study showed that fungal communities differed significantly by management and sampling position; bacterial communities were also strongly influenced by spatial position, with distinguishable structures between tree rows and inter-row cover strips [104]. Such increased spatial heterogeneity may maintain higher diversity and, via functional redundancy, enhance stability and recovery under disturbance [105].

### 4.2. Improved soil physicochemical properties and optimization of microbial habitats

Cover cropping can improve soil physicochemical properties through multiple pathways, providing more suitable habitats and substrate sources for microbes. Structurally, cover crops often reduce bulk density, improve pore architecture, and promote infiltration and aeration, with more pronounced effects under sustained multi-year coverage [106]. These improvements are closely associated with the formation and stabilization of macroaggregates, commonly reflected by increases in the proportion of water-stable aggregates (>0.25 mm) and higher mean weight diameter (MWD), which enhance structural stability and resistance to erosion while improving water retention [107],[108]. Such structural optimization offers microbes more attachment surfaces, more favorable air–water configurations, and more continuous diffusion pathways, benefiting aerobic microbial activities and rhizosphere processes in particular [106]. In terms of nutrients and biological processes, cover crops increase SOC and labile substrates via rhizodeposition and residue return, which in turn stimulates microbial biomass, alters community structure and functional guilds, and strengthens microbially mediated nutrient transformations [106],[109]. Meta-analyses at the scale of Chinese orchards indicate that groundcover management can increase activities of enzymes involved in C, N, and P acquisition and oxidative decomposition by ~14%–37%, suggesting enhanced microbial metabolism and nutrient cycling [110]. Changes in soil pH and nutrient-retention capacity further influence microbial physiology and community composition. In acidic orchard soils, cover crops are often associated with pH recovery or mitigation of acidification, though effects vary with cover type, duration, fertilization regime, and environmental conditions [110],[111]. Mechanistically, surface cover reduces erosion and losses of nutrients/base cations, promotes base-cation recycling via biomass return, and increases organic matter buffering and charge sites, thereby stabilizing or increasing cation exchange capacity (CEC) and nutrient-retention capacity, providing a more stable nutrient supply to microbes [112].

Cover crops also regulate soil water status by improving soil structure and hydraulic properties, thereby affecting microbial activity and community composition. In general, cover cropping can reduce bulk density, increase macroporosity and aggregate stability, and enhance infiltration and saturated hydraulic conductivity, facilitating the infiltration of rainfall/irrigation water and improving soil water storage and plant-available water under many scenarios [113]. However, under water limitation or prolonged drought, living groundcovers may compete with trees for water, decreasing soil moisture and constraining or shifting microbial metabolism and community composition. In a 15 year red-soil citrus orchard experiment in southern China, Tu et al. reported that groundcover (bahiagrass and bermudagrass) reduced runoff and erosion and gradually improved topsoil properties; however, during the dry season (August–September), soil moisture in 0–40 cm was highest under clean tillage and lowest under full-surface cover, and yield reductions due to lower water availability were more pronounced under full cover than strip cover, highlighting the importance of cover patterns in water-competition risk [114]. Long-term apple orchard studies have also shown that cover crops (*Dactylis glomerata* L., *Trifolium repens*, *Coronilla varia* L., *Lotus corniculatus* L.) can significantly reduce soil moisture in spring and summer and are associated with seasonal changes in microbial carbon-metabolic profiles, which have been attributed to tree-cover water competition [115]. Conversely, cover crops do not always reduce soil moisture or microbial performance. In a pecan orchard in California, USA, inter-row cover crops (*Trifolium incarnatum*, *T. pratense*, *T. resupinatum*, and *Avena sativa*) did not deplete soil water or nutrients; instead, they often increased soil moisture and microbial biomass and favored copiotrophic communities, indicating strong regulation by climate, water supply, and management [33]. Species differences in root density and water-use strategies are key determinants of whether cover crops improve water retention or intensify competition [108]. In irrigated Mediterranean vineyards in semi-arid regions, *Phacelia tanacetifolia* and rye (*Secale cereale* L. ‘Merced’) cover increased under-vine soil moisture and enhanced vine vigor, suggesting that species selection and management can reduce water-competition risks while retaining ecological benefits [116]. Climate-change scenario experiments have further shown that reduced precipitation can shift the direction and magnitude of cover-crop effects on microbial network complexity and soil functions, underscoring the climate sensitivity of the cover–water–microbiome nexus [117].

### 4.3. Reconstruction of the soil micro-food web and cascading effects on nutrient cycling

Biological effects of cover cropping extend beyond bacteria and fungi and may restructure the soil micro-food web composed of microorganisms and microfauna (e.g., protists and nematodes), thereby altering energy flow and nutrient-cycling pathways. Multi-trophic syntheses emphasize that soil food webs, comprising bacteria, fungi, protists, nematodes, and other groups, are coupled by bottom-up and top-down controls and sustain ecosystem functioning and stability. Among them, bacterial, fungal, and root energy channels are key pathways for energy transfer [118]. By increasing rhizodeposition and litter inputs and improving water and nutrient conditions, cover crops often alter the composition of nematode and other trophic groups and their food-web structure. In kiwifruit orchards with multi-species covers, nematode community structure and network complexity increased; footprint-based analyses indicated enhanced energy flow and improved stability-related indices, and soil moisture, SOC, nitrate N, pH, microbial biomass, and cover-crop biomass were identified as key drivers [119]. In a California Mediterranean-climate almond orchard, Wauters et al. compared natural vegetation dominated by *Vicia*, *Malva*, and *Hordeum* with winter cover-crop mixtures that were functionally diverse (*Sinapis alba*, *Raphanus sativus*, *Trifolium alexandrinum*, *Vicia sativa*, and *Secale cereale*) and functionally simple (*Brassica napus*, *B. juncea*, *S. alba*, and *R. sativus*). They found that cover-crop community composition altered nematode food-web complexity (e.g., Structure Footprint), and nematode shifts were primarily driven by cover-crop biomass and microbial biomass N (MBN), indicating that different species combinations can rapidly reshape soil biological networks within a single season through coupled *biomass inputs–microbial N pool* dynamics [120]. In row-crop systems (corn–soybean) with winter covers, Akanwari et al. reported that long-term cover with rye (*Secale cereale*), barley (*Hordeum vulgare*), and oat (*Avena sativa*) increased nematode abundance and diversity and raised structural and enrichment indices, which are often interpreted as indicators of more active nutrient cycling and a more mature/stable food web [121].

Food-web restructuring can also feed back to microbial communities and functions. Protists, as major consumers in the micro-food web, can reshape bacterial community structure through selective grazing and enhance nutrient remineralization via the microbial loop. Recent mechanistic studies further suggest that protists can act as *transport vectors* that carry beneficial bacteria to growing root tips, altering spatial distributions of rhizosphere microbes and plant benefits [122],[123]. Nematodes span multiple trophic levels (bacterivores, fungivores, predators/omnivores), and changes in nematode assemblages influence the relative importance of microbial energy channels and the efficiency of energy transfer, producing cascading effects on decomposition, nutrient release, and plant–soil feedback. Therefore, incorporating a *multi-trophic–network structure–functional stability* framework often explains soil-health and resilience differences better than focusing only on microbial composition [118],[124].

**Figure 3.**
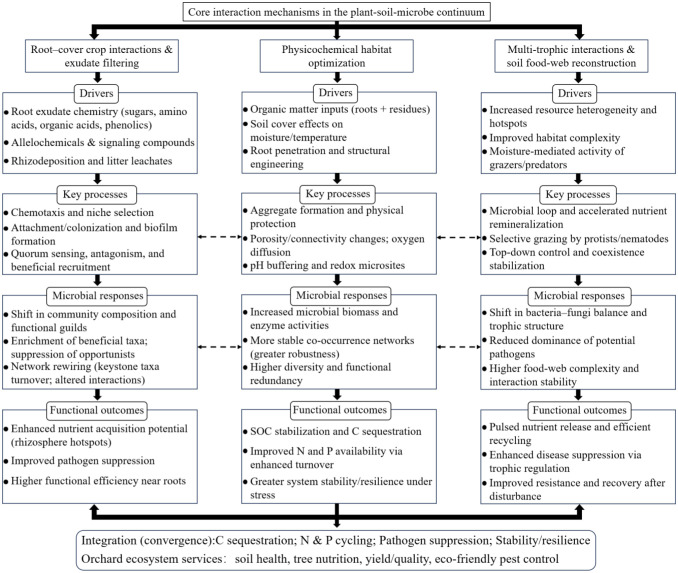
Parallel mechanisms linking orchard cover cropping to microbial community reassembly and functional outcomes. Note: Cover cropping influences soil microbiomes through three interacting pathways: (i) root–cover crop interactions and exudate filtering, which drive rhizosphere habitat filtering and selective recruitment; (ii) physicochemical habitat optimization, whereby organic matter inputs and microclimate regulation improve aggregation/porosity and create favorable pH/redox microsites; and (iii) multi-trophic interactions and soil food-web reconstruction, in which protist/nematode grazing and top-down control reshape trophic structure and nutrient remineralization. Across pathways, cascading effects from drivers to key processes, microbial responses (including shifts in functional guilds and network properties), and functional outcomes converge on enhanced C sequestration, N and P cycling, pathogen suppression, and stability/resilience. Context dependencies (plant traits, cover duration, soil depth, climate, and fertilization regime) modulate pathway strength and direction. Solid arrows indicate primary causal pathways; dashed arrows indicate cross-talk among pathways.

Cover crops also affect larger soil fauna by modifying surface soil moisture and temperature and by increasing organic inputs; in some cases, pH changes may also contribute, leading to short-term shifts in earthworms, collembolans, mites, and nematodes that further reshape food-web structure and function [119],[125]. In a two-year field trial in commercial apple orchards in the UK, Webber et al. showed that transferring mowed biomass from inter-row legume–grass covers (*Medicago sativa*, *Trifolium pratense*, *Dactylis glomerata*, and *Phleum pratense*) to the in-row herbicide strip significantly increased topsoil moisture. Under the double-return treatment, earthworm abundance and biomass increased by ~1.7 and ~1.8-fold, respectively, relative to the control [125]. In vineyards, Vršič et al. likewise reported that soil moisture is a key driver of earthworm fluctuations, with earthworm biomass declining in drought years [126]. Groundcover type can also markedly affect collembolan communities, and in kiwifruit orchards, cover crops can regulate nematode food-web energy flow through changes in soil moisture and pH [119]. Over longer timescales, the stimulatory effects of cover cropping/organic management and increased plant diversity on soil fauna tend to strengthen with time [118]. Mechanistically, earthworm burrowing, mixing, and casting promote aeration and infiltration, aggregate formation, and nutrient availability [127]. Small- and medium-sized soil fauna accelerate decomposition and nutrient turnover by fragmenting residues and regulating microbial metabolism, thereby providing positive feedback to soil health [128]. The interacting pathways through which cover cropping reshapes the plant–soil–microbiome continuum are conceptually summarized in Figure 3.

## 5. Challenges and perspectives

Overall, existing studies demonstrate that cover cropping reshapes orchard soil microbiomes, but three major gaps remain. First, many experiments are short-term, limiting our ability to characterize steady-state responses and interannual variability of microbiomes and ecosystem functions. Second, studies often emphasize community composition over function and mechanisms, relying largely on correlational interpretations and lacking causal evidence from functional gene expression, metabolic processes, and element flux measurements. Third, sites and climate zones are unevenly represented: evidence from temperate orchards is relatively abundant, whereas subtropical and tropical hilly orchards differ markedly in hydrothermal regimes and soil backgrounds, and the transferability of conclusions requires further testing. Addressing these limitations will require a shift from descriptive pattern recognition toward stronger causal inference. In particular, the combined use of metagenomics, metatranscriptomics, metabolomics, stable-isotope tracing, and process-based measurements will make it possible to determine not only which microorganisms are present but also which groups are active, what substrates they utilize, and how their activities contribute to carbon stabilization, nutrient turnover, and disease suppression. Quantifying microbial necromass formation, carbon-use efficiency, and the coupling between functional genes and biogeochemical fluxes will be especially important for resolving the mechanistic roles of cover crops in orchard soils.

Future work should advance along the structure–function–service pathway: (1) build long-term experimental platforms and integrate metagenomics/metatranscriptomics/metabolomics (and, when needed, stable-isotope probing) to connect functional genes with process fluxes; (2) develop causal models linking management–soil habitat–microbiome–tree health/quality (e.g., structural equation models) and quantify contributions of key pathways; (3) conduct cross-regional and cross-soil comparisons to identify transferable mechanisms and context dependence; and (4) translate microbiome knowledge into practice by selecting cover species/mixtures for scenarios characterized by acidification, disease pressure, and water limitation, and exploring synergistic management such as cover cropping + microbial inoculants to enhance soil health and system stability.

## 6. Conclusions

Cover cropping is a sustainable soil-management strategy for hilly and mountainous orchards that can substantially influence the structure and function of the soil microbiome through multiple pathways. Across the literature synthesized here, cover cropping consistently alters bacterial and fungal community structure, increases microbial diversity, and enhances the complexity and stability of microbial networks; these effects vary by cover species, with legumes tending to promote N-cycling guilds whereas grasses more strongly stimulate C-cycling decomposers. Cover cropping also strengthens microbially mediated carbon cycling and nutrient mobilization, increasing SOC stabilization potential and improving nutrient-use efficiency, while enriching beneficial taxa and suppressing soil-borne pathogens, thereby enhancing ecosystem services and tree health. These outcomes are underpinned by coupled mechanisms involving tree–groundcover interactions driven by root exudates, improvements in soil physicochemical habitats, and multi-trophic interactions within the soil food web. With advances in multi-omics integration and the establishment of long-term field trials, future research will further resolve causal mechanisms and support the development of microbiome-informed, scenario-specific groundcover strategies, providing actionable indicators and pathways for cover species selection, spatial cover-pattern optimization, fertilizer reduction with improved efficiency, and disease mitigation with improved fruit quality.

## Use of AI tools declaration

The authors declare they have not used Artificial Intelligence (AI) tools in the creation of this article.
